# Mps1 inhibitors synergise with low doses of taxanes in promoting tumour cell death by enhancement of errors in cell division

**DOI:** 10.1038/s41416-018-0081-2

**Published:** 2018-05-08

**Authors:** Ana Rita R. Maia, Simon Linder, Ji-Ying Song, Chantal Vaarting, Ute Boon, Colin E. J. Pritchard, Arno Velds, Ivo J. Huijbers, Olaf van Tellingen, Jos Jonkers, René H. Medema

**Affiliations:** 1 Division of Cell Biology and Cancer Genomics Center, The Netherlands Cancer Institute, Plesmanlaan 121, Amsterdam, 1066 CX Netherlands; 2 Division of Experimental Animal Pathology, The Netherlands Cancer Institute, Plesmanlaan 121, Amsterdam, 1066 CX Netherlands; 3 Division of Molecular Pathology and Cancer Genomics Center, The Netherlands Cancer Institute, Plesmanlaan 121, Amsterdam, 1066 CX Netherlands; 4 Transgenic Core Facility, Mouse Clinic for Cancer and Aging (MCCA), The Netherlands Cancer Institute, Plesmanlaan 121, Amsterdam, 1066 CX Netherlands; 5 Genomics Core Facility, The Netherlands Cancer Institute, Plesmanlaan 121, Amsterdam, 1066 CX Netherlands; 6 Laboratory of Clinical Chemistry and Hematology, The Netherlands Cancer Institute, Plesmanlaan 121, Amsterdam, 1066 CX Netherlands; 7 Present Address: Division of Oncogenomics, The Netherlands Cancer Institute, Plesmanlaan 121, Amsterdam, 1066 CX Netherlands

**Keywords:** Cell division, Cancer therapy

## Abstract

**Background:**

Chromosomal instability (CIN) is a common trait of cancer characterised by the continuous gain and loss of chromosomes during mitosis. Excessive levels of CIN can suppress tumour growth, providing a possible therapeutic strategy. The Mps1/TTK kinase has been one of the prime targets to explore this concept, and indeed Mps1 inhibitors synergise with the spindle poison docetaxel in inhibiting the growth of tumours in mice.

**Methods:**

To investigate how the combination of docetaxel and a Mps1 inhibitor (Cpd-5) promote tumour cell death, we treated mice transplanted with BRCA1^−/−^;TP53^−/−^ mammary tumours with docetaxel and/or Cpd-5. The tumours were analysed regarding their histopathology, chromosome segregation errors, copy number variations and cell death to understand the mechanism of action of the drug combination.

**Results:**

The enhanced efficacy of combining an Mps1 inhibitor with clinically relevant doses of docetaxel is associated with an increase in multipolar anaphases, aberrant nuclear morphologies and cell death. Tumours treated with docetaxel and Cpd-5 displayed more genomic deviations, indicating that chromosome stability is affected mostly in the combinatorial treatment.

**Conclusions:**

Our study shows that the synergy between taxanes and Mps1 inhibitors depends on increased errors in cell division, allowing further optimisation of this treatment regimen for cancer therapy.

## Introduction

Chromosomal instability (CIN) is a common trait of human cancer, found in more than 40% of solid tumours.^1^ While the gain or loss of an entire chromosome results in numerical CIN, the inheritance of broken chromosomes can lead to chromosomal translocations, referred to as structural CIN. Regardless of its classification, the causes of CIN have been attributed to spindle assembly checkpoint (SAC) deficiencies, improper stability of the kinetochore-microtubule attachments, defects in sister chromatid cohesion, supernumerary centrosomes and replication stress.^2^ At the cellular level, CIN is characterised by the continuous gain and loss of chromosomes during cell division. In tissues, CIN has been more difficult to monitor due to the challenges associated with the analysis of the fidelity of cell division in situ. Therefore, gene expression signatures, nuclear grading, flow cytometry, fluorescence in situ hybridisation and other genomic approaches have been used as surrogate read-outs of CIN status.^1^

CIN leads to the generation of de novo aneuploidies, not all of which will confer a selective advantage. In fact, aberrant chromosome segregation can be associated with mitotic catastrophe, a type of cell death that occurs during mitosis.^3^ Moreover, it is well established that aneuploidy generally has a detrimental effect on cell proliferation and viability, but the genetic background of the cell and the nature of the aneuploidy can confer a proliferative advantage.^2^ This means that many of the newly generated aneuploidies will result in a block in further proliferation, while only rare selected cases will produce a growth advantage. The effect of CIN on tumour fitness is a matter of balance; induction of a low/moderate level of CIN can promote tumour formation, but high levels of CIN appear to have a tumour suppressive effect.^4^ This is consistent with studies in breast, ovarian, gastric and non-small-cell lung cancer, in which patients with high levels of CIN have a better prognosis than patients with a low level of CIN.^5,6^ These observations imply that enhancement of CIN could be a useful therapeutic strategy. In fact, conventional chemotherapeutic agents like taxanes induce chromosome segregation errors.^7^ Similarly, the Mps1/TTK kinase has been exploited as a potential therapeutic target to induce CIN. Mps1 is a kinase with a key role in the establishment of the SAC signalling and in the correction of erroneous kinetochore-microtubule attachments. Inhibition of Mps1 can induce overt segregation errors^8^ and Mps1 is overexpressed in a wide variety of tumours.^9^ Several small-molecule inhibitors of Mps1 kinase have been characterised in vitro and in vivo.^10–32^ In addition, the combination of Mps1 inhibitors with low doses of taxanes act synergistically in promoting tumour cell death in both tissue culture and murine tumours.^14,25,30,32,33^ In murine tumours, the drug combination elevates the amount of cell death^14^ and induces nuclear pleomorphism.^14,25,32^ Based on these promising preclinical results, three phase I clinical trials have been initiated using Mps1 inhibitors in combination with paclitaxel.^34–36^ However, the mode of action by which the drug combination induces tumour cytotoxicity in vivo has not been resolved. Here we show that the combination of Mps1 inhibitors and docetaxel leads to tumour cell death by elevating the levels of CIN, as evidenced by an increase in multipolar cell divisions, enhanced chromosome copy number variations and an increase in nuclear pleomorphism in BRCA1^−/−^;TP53^−/−^ mammary tumours.

## Materials and methods

### Compounds and drugs

Cpd-5 was synthesised according to patent WO 2009156315A1 from Nerviano Medical.^10^ The synthesis, structure and activity has been published previously in ref.^15^. Cpd-5, paclitaxel (Sigma) and BAY-1217389 (Cayman Chemical) were dissolved in dimethyl sulphoxide (DMSO). Docetaxel (Accord) was diluted in saline, whereas Cpd-5 was diluted in vehicle (5% DMSO, 5% cremophor, 5% mannitol).

### Cell culture

KB1P-B11^37^ and KP3 cells^38^ were grown in Dulbecco's modified Eagle's medium/F-12 (Fisher Scientific), supplemented with 10% foetal calf serum (Clontech), 50 μg/mL penicillin–streptomycin (Invitrogen), 5 μg/mL insulin (Sigma), 5 ng/mL epidermal growth factor (PeproTech) and 5 ng/mL cholera toxin (Sigma). MCF10A, MCF7, MDA-MB-361, MDA-MB-468, MDA-MB-231 and SK-BR-3 cell lines were purchased form the American Type Culture Collection and cultured according to the recommendations. All cells were grown at 37 °C in a humid atmosphere with 5% CO_2_.

### Intervention studies

Tumours were collected from the *K14cre;Brca1*^*f/f*^*;tp53*^*f/f*^ female mice^39^ and cryopreserved. Orthotopic transplantation of BRCA1^−/−^;TP53^−/−^ tumours in wild-type FVB/NrJ mice was performed as previously described.^40^ The tumour volume was monitored at least three times a week by caliper measurements and calculated with the formula: 0.5 × length x width^2^. When tumours reached a size of approximately 200 mm^3^, animals were treated with different docetaxel doses (25 and 12.5 mg/kg, once every week intravenously), Cpd-5 (5 and 10 mg/kg, once every other day intraperitoneally (i.p.)) or vehicle (once every other day i.p.). Docetaxel treatments were interrupted if tumours regressed to less than 50% of initial size and resumed when tumours relapsed to 100% of start size. Vehicle and Cpd-5 treatments took place during 28 days. Whenever the tumours did not regress to 50% of initial size, Cpd-5 treatments were continued for 28 more days. Animals were killed by CO_2_ asphyxiation in case of signs of drug toxicity or if tumours reached a maximum size of 1500 mm^3^. The Animal Ethics Committee of the Netherlands Cancer Institute approved all animal experiments.

### Additional materials and methods

Description of study design and materials and methods used for cell proliferation assays, flow cytometry-based cell cycle analysis, live cell imaging, chromosome spreads, CRISPR/Cas9-mediated genome editing, genotyping, histopathology, copy number variation sequencing, pharmacokinetic studies and statistical analysis can be found in the Supplementary Materials and methods section.

## Results

### Cpd-5 and paclitaxel synergise to induce mitotic errors and tumour cell death in vitro

**Fig. 1:**
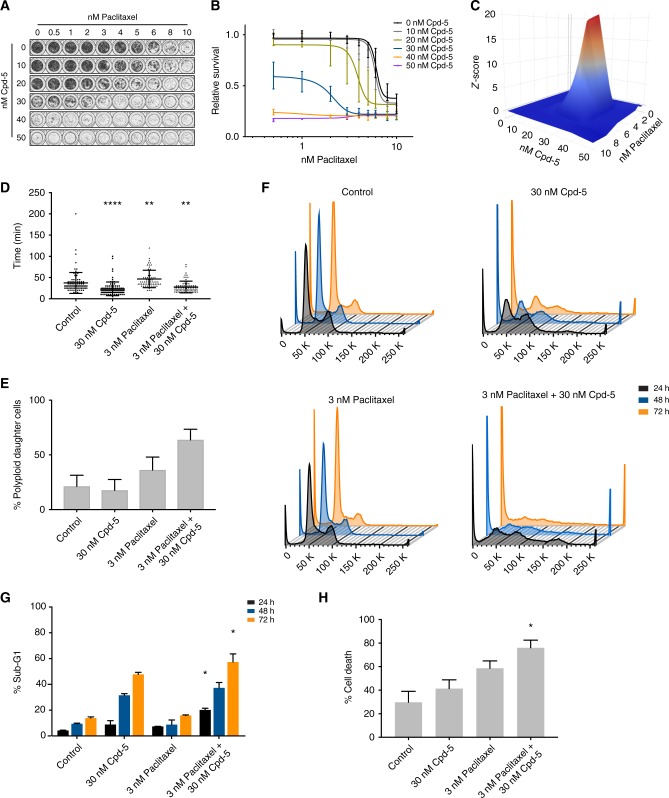
Paclitaxel and Mps1 inhibitors have a synergistic cytotoxic effect in BRCA1^−/−^;TP53^−/−^ tumour cell lines. **a** Representative colony formation assay of KB1P-B11 cells treated with paclitaxel and/or Cpd-5. **b** Relative survival plots of paclitaxel-treated cells with and without Cpd-5. Curves represent the average and standard deviations (*n* = 3). **c** 3D synergy plots of the drug combination between Cpd-5 and paclitaxel. **d** Scatter dot plot representation of time in mitosis (from NEB to anaphase onset) of KB1P-B11 cells untreated (control), treated with 30 nM Cpd-5, 3 nM paclitaxel or with the drug combination. Bars represent the mean and standard deviation (SD). Time in mitosis was 37.3 ± 24.6 min (mean ± SD) for untreated cells (*n* = 89), 23.4 ± 13.0 min for cells treated with 30 nM Cpd-5 (*n* = 87), 46.7 ± 20.5 min for cells treated with 3 nM paclitaxel (*n* = 63), and 27.7 ± 13.7 for cells treated with the combination (*n* = 73). Means are statistically different (asterisks), *A* < 0.05 (Dunn’s test). **e** Percentage of polyploid cells quantified in (**c**). Bars represent the percentage of polyploid cells in the total population, error bars represent the 95% confidence intervals (C.I.). **f** Flow cytometric cell cycle analysis of KB1P-B11 cells untreated or treated with 30 nM Cpd-5, 3 nM paclitaxel or a combination of both for 24, 48 or 72 h. **g** Percentage of sub-G1 population quantified from the plots in (**f**) (*n* = 2). **h** Quantification of cell death induced after 72 h of the indicated drug treatments assessed by live cell imaging. Bars represent the mean and error bars the SD, statistical differences relative to the control sample are signed with an asterisk, *A* < 0.05 (Dunn’s test)

Combining taxanes and Mps1 inhibitors extends the survival of mice bearing BRCA1^−/−^;TP53^−/−^ tumours,^25^ but the mechanism underlying this synergy remains unknown. As a first approach, we treated an established cell line from this tumour model, KB1P-B11,^37^ with increasing concentrations of paclitaxel, with or without Cpd-5 (Fig. 1a). In the presence of Cpd-5, the KB1P-B11 cells became more sensitive to paclitaxel (Fig. 1b), resulting in a synergistic interaction (Fig. 1c) and consequent reduction of half-maximal inhibitory concentrations (IC_50_s) of paclitaxel (Table S1). To pinpoint whether this synergy is restricted to Cpd-5, we treated the KB1P-B11 with BAY-1217389,^32^ a Mps1 inhibitor currently in clinical trial.^35,36^ Similarly to Cpd-5, we observed that the co-treatment with paclitaxel and BAY-1217389 resulted in a decrease of paclitaxel IC_50_ (Fig S1A). Thus, the synergistic toxicity of paclitaxel and Mps1 inhibitors is BRCA1^−/−^;TP53^−/−^ tumour cell intrinsic.

Next, we tested whether this synergy could also be observed in other breast cancer cell lines. To this end, we treated a panel of breast cancer cell lines arising from different tumour subtypes and genotypes (Table S2) and analysed the effect of combining paclitaxel and Cpd-5 in cell survival. We observed a heterogeneous response to the drug combination (Fig. S1B and Table S1), in which the KB1P-B11 and MDA-MB-231 were the ones with the highest synergy scores, and MCF7 and SK-BR-3 the most refractory (Table S3). We scored the base level of chromosome missegregations (Fig. S1C), the expression levels of Mps1 (Fig. S1D) in order to predict if any of these factors confer sensitivity to the drug combination. However, we could not observe any significant correlation between these two parameters and the synergistic effect between Cpd-5 and docetaxel (Fig. S1E). However, the higher levels of Mps1 expression corresponded to a higher resistance to paclitaxel (Fig. S1F), as previously described.^41^

Next, we analysed the effect of these drugs on the fidelity of cell division by live cell imaging. The treatment with 30 nM of Cpd-5 reduced the time KB1P-B11 cells spent in mitosis from 37 min (from nuclear envelope breakdown (NEB) to anaphase) to 23 min (Fig. 1d). A clinically relevant concentration of paclitaxel (3 nM) resulted in a transient mitotic arrest (47 min) that could be reverted by the addition of 30 nM of Cpd-5 (28 min). By live cell imaging, we observed a high number of cells treated with the drug combination that underwent cytokinesis failure or mitotic slippage events (Fig. S2A, B), which resulted in polyploid cells (Fig. 1e). Exposure to Cpd-5 caused the ploidy of the cells in the population to become more variable, as evidenced by a broadening of the 4',6-diamidino-2-phenylindole (DAPI) peaks (Fig. 1f) and resulted in a higher percentage of cells with a sub-G1 DNA content (Fig. 1g). The treatment with paclitaxel alone had no apparent impact on the cell cycle distribution (Fig. 1f), indicating that the concentration of paclitaxel used in these experiments is insufficient to trigger a prominent mitotic arrest. However, the combination of paclitaxel and Cpd-5 induced a dramatic variation in DNA content (Fig. 1f) and resulted in more cell death (Fig. 1g) after 48 and 72 h of drug treatment. The enhanced cytotoxic effect of the drug combination was also observed by imaging the cells in the presence of TOPRO to visualise cell death (Fig. 1h). Consistent with these observations, karyotyping of the KB1P-B11 cells showed that treatment with paclitaxel and Cpd-5 induced a clear increase in aneuploidy (Fig. S2C). Thus, the combination of paclitaxel and Cpd-5 induces severe aneuploidy and cell death in the KB1P-B11 cell line.

### Cpd-5 exerts in vitro cytotoxic effects through Mps1 kinase inhibition

**Fig. 2:**
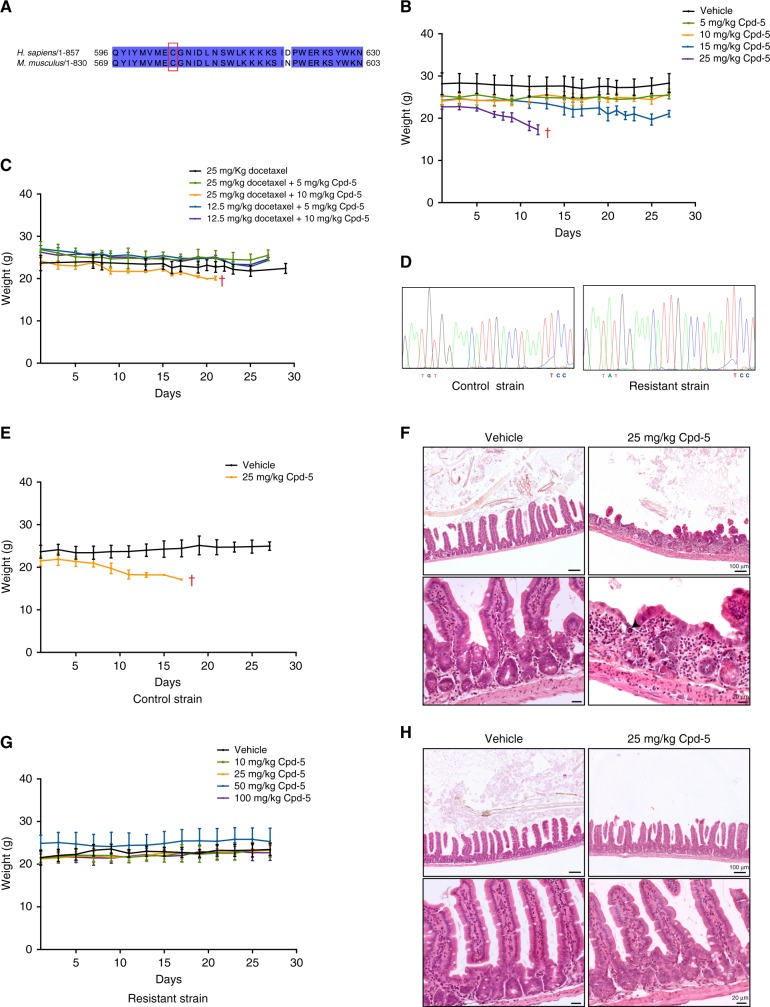
In vivo target engagement of Compound 5. **a** Protein alignment of human and murine Mps1 kinase sequences. Conserved aminoacids are highlighted in blue, red box underlines cysteine in residue 604 and 577 in humans and mice, respectively. **b** Maximum tolerated studies in FVB/NrJ wild-type mice treated with increasing doses of Cpd-5 (*n* = 5 mice per condition). Curves represent the average weight and standard deviation. **c** Maximum tolerated studies in FVB/NrJ wild-type mice treated with different concentrations of docetaxel and Cpd-5 (*n* = 5 mice per condition). Curves represent the average weight and respective standard deviation. **d** Genotype of control and resistant mice, mutated residues are highlighted. **e**,** g** Maximum tolerated studies in control mice treated with vehicle and 25 mg/kg Cpd-5 (**e**), or resistant mice treated with vehicle and increasing doses of Cpd-5 (**g**). Curves represent the average weight and the standard deviation. The symbol '†' marks treatment groups killed due to severe weight loss. **f**,** h** Representative images of H&E stainings of the ileum of control (**f**) and resistant (**h**) mice treated with vehicle (left panel) and 25 mg/kg Cpd-5 (right panel). Lower panels show higher magnification examples of the images depicted in the upper panels

One of the biggest challenges regarding the validation of new small-molecule inhibitors is the in vivo confirmation of target engagement. To confirm that the cytotoxic effects of Cpd-5 are due to inhibition of Mps1, we edited the genome of the KB1P-B11 cell line with CRISPR/Cas9 to introduce a Cpd-5-resistant mutation (C577Y corresponding to C604 in humans)^15^ (Fig. 2a). Cell proliferation assays showed a more than 30-fold resistance of C577Y-edited cells to Cpd-5 in comparison to wild-type cells (Fig. S3A). Importantly, the drug synergy between paclitaxel and Cpd-5 was completely abolished in the KB1P-B11/C577Y cell line (Fig. S3B–D, Table S4), indicating that these effects are achieved through inhibition of the murine Mps1 kinase by Cpd-5.

### Inhibition of Mps1 in mice by Cpd-5

The range of concentrations and the cytotoxicity induced by Cpd-5 in vivo were assessed by maximum tolerated dose (MTD) studies in FVB/NrJ wild-type mice. A dose of 10 mg/kg is the MTD of Cpd-5 in mice and higher doses of Cpd-5 (≥15 mg/kg) lead to weight loss (Fig. 2b), similar to other Mps1 inhibitors.^14,25^ To determine whether this toxicity is enhanced by co-treatment with a taxane, we selected docetaxel since this is used in the clinic, and the relevant concentrations have been determined.^40^ Combination of 10 mg/kg of Cpd-5 with the full MTD of docetaxel (25 mg/kg) led to significant weight loss, which was not observed if lower concentrations of docetaxel or Cpd-5 were used (Fig. 2c). We therefore set the working concentrations for the drug combination as 25 mg/kg of docetaxel with 5 mg/kg of Cpd-5, and 12.5 mg/kg of docetaxel in the presence of the full MTD of Cpd-5.

To address if the cytotoxic effects of Cpd-5 were due to inhibition of Mps1, we used a mouse strain expressing the C577Y mutation (Fig. 2d) to investigate the resistance to Cpd-5. Control mice treated with 25 mg/kg of Cpd-5 presented weight loss (Fig. 2e) with severe reduction of the intestinal crypts (Fig. 2f), accompanied with apoptosis and impaired haematopoiesis in the bone marrow and spleen (Fig. S4A, Table S5). Strikingly, all of these toxicities were absent in the C577Y-expressing mice treated with doses up to 100 mg/kg of Cpd-5 (Fig. 2g, h, S4B). Thus, the toxic effects of Cpd-5 in mice appear to be entirely caused by inhibition of Mps1 kinase, making it an ideal drug for intervention studies.

### Docetaxel and Cpd-5 improve the survival of mice transplanted with BRCA1^−/−^;TP53^−/−^ tumours

**Fig. 3:**
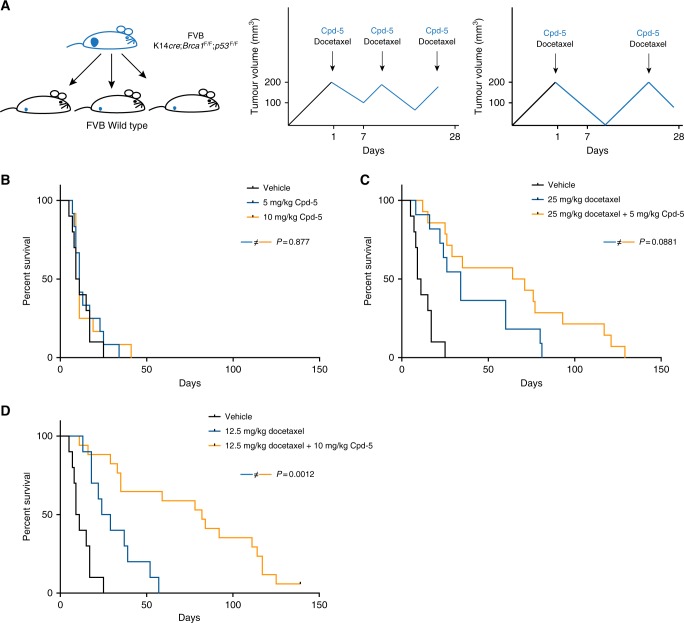
Increased survival of mice transplanted with BRCA1^−/−^;TP53^−/−^ mammary tumours upon treatment with Compound 5 and docetaxel. **a** Schematic representation of tumour transplantation (left panel) and dosing schemes (middle and right panels). Black arrows represent docetaxel treatments; arrows and continuous blue lines correspond to treatment with Cpd-5 every other day. **b** Survival plots of mice treated with vehicle, 5 or 10 mg/kg Cpd-5 (*n* = 10, 11 and 11, respectively). **c** Survival plots of mice treated with vehicle, 25 mg/kg docetaxel alone or in combination with 5 mg/kg Cpd-5 (*n* = 10, 11 and 14, respectively). **d** Survival plots of mice treated with vehicle, 12.5 mg/kg docetaxel alone or in combination with 10 mg/kg Cpd-5 (*n* = 10, 10 and 17, respectively). The log-rank *p* values are indicated

To study the effect of combining Cpd-5 and a taxane in promoting tumour regression, we transplanted FVB/NrJ wild-type mice with K14cre;BRCA1^−/−^;TP53^−/−^ tumours^39^ and treated the tumours with vehicle, 5 or 10 mg/kg of Cpd-5, 12.5 or 25 mg/kg of docetaxel, 25 mg/kg of docetaxel with 5 mg/kg of Cpd-5 or 12.5 mg/kg of docetaxel with 10 mg/kg Cpd-5 (Fig. 3a). The groups treated with vehicle or Cpd-5 alone showed no differences in tumour growth (Fig. S5A) or overall survival (Fig. 3b). The treatment with 12.5 mg/kg or 25 mg/kg of docetaxel increased the median survival of the mice from 10 (vehicle) to 26 or 34 days, respectively (Fig. 3c, d). Combination with 5 mg/kg or 10 mg/kg of Cpd-5 increased the median survival to 68 or 82 days, respectively (Fig. 3c, d). This clear and statistically significant improvement in survival is a reflection of more sustained shrinkage cycles in the tumours after treatment with the drug combination (Fig. S5B,C). Strikingly, one of the treated tumours from the combinatorial regimen never relapsed and there were only a few non-dividing tumour cells left in the mammary fat pad (Fig. S5D). Taken together, these data show that the combination of docetaxel and Cpd-5 acts synergistically in the treatment of mice bearing BRCA1^−/−^;TP53^−/−^ tumours.

### Combining docetaxel and Cpd-5 induces cellular pleomorphism and CIN

**Fig. 4:**
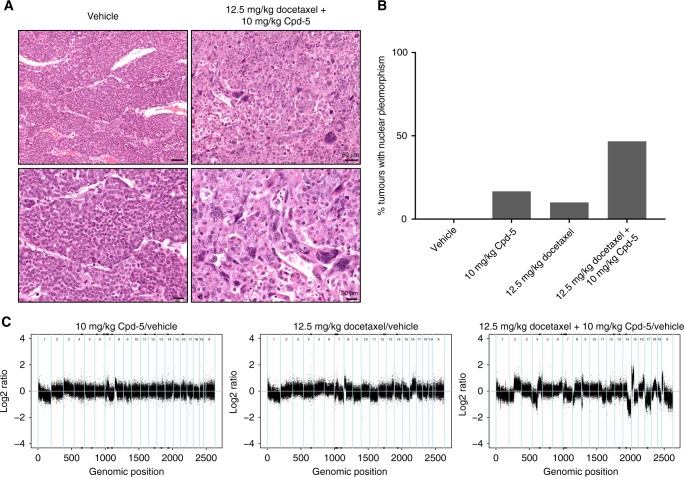
Combination of Mps1 inhibitors and docetaxel induces cellular pleomorphism. **a** Representative images of tumour tissue sections stained with H&E treated with vehicle (left panel) and 12.5 mg/kg docetaxel and 10 mg/kg Cpd-5 (right panel). Lower panels show higher magnification examples of the images depicted in the upper panels. **b** Percentage of nuclear pleomorphism observed in the treated tumours scored by histopathological analysis (*n* ≥ 10 tumours per condition). **c** Normalized copy number variation profiles of the treated tumour samples. The profiles were normalised by subtracting the copy number variation of the vehicle-treated tumour to the ones treated with Cpd-5 and/or docetaxel

Based on data obtained in cultured cell lines, we anticipated that the synergistic effect of docetaxel and Cpd-5 stems from enhanced cell division errors in the tumours treated in mice. Given the strong synergy observed at 12.5 mg/kg of docetaxel and 10 mg/kg of Cpd-5, we first analysed their effect on tumours that were collected at the end-point of treatment (tumour ≥1500 mm^3^). Although these tumours are no longer sensitive to the drug treatment, we could observe that the tumours treated with Cpd-5, docetaxel or both drugs (but not the vehicle-treated) displayed an increase in nuclear pleomorphism (heterogeneous nuclear size and morphology) (Fig. 4a, b), a common readout of CIN.^42^ Most importantly, nuclear pleomorphism was approximately fivefold higher in the samples treated with the combination of 12.5 mg/kg docetaxel and 10 mg/kg of Cpd-5, compared to treatment with docetaxel alone.

To directly address the amount of CIN induced, we performed copy number variation (CNV) sequencing in these tumour samples. The vehicle-treated tumour displayed several copy number changes (Fig. S6), in accordance to what has been described for the BRCA1^−/−^;TP53^−/−^.^39^ To visualise the copy number changes that these tumours underwent upon treatment with docetaxel and/or Cpd-5, we subtracted the vehicle CNV from the CNV pattern obtained in the treated tumours (Fig. 4c). We observed that Cpd-5 and docetaxel as single agents induced minor changes in CNV, but the drug combination caused more dramatic changes in chromosome copy number, supporting our hypothesis that we are perturbing the chromosome content of the tumours. These results imply that the drug combination induces a higher level of CIN compared to the single treatments.

### Co-administration of docetaxel and Cpd-5 leads to more multipolar cell divisions and cell death

**Fig. 5:**
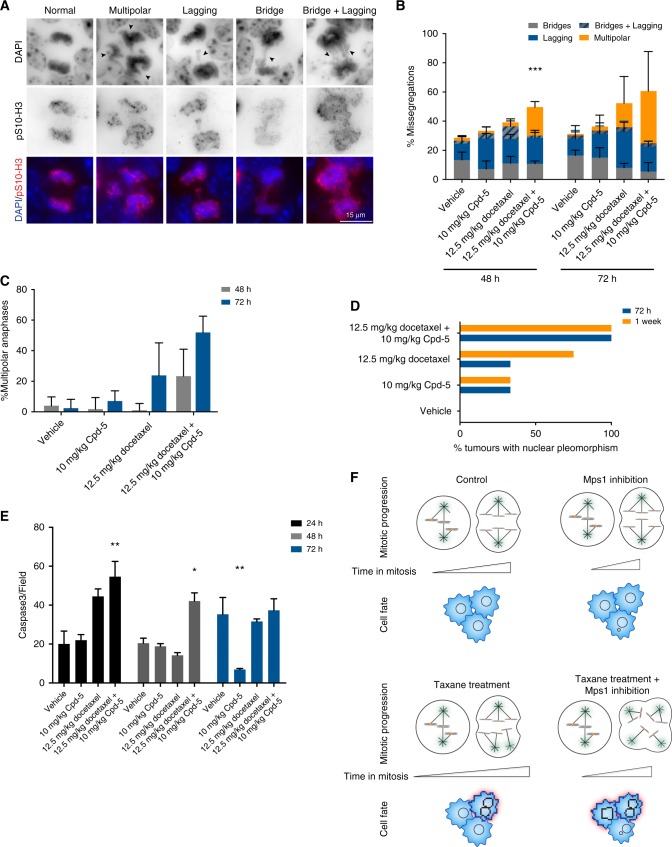
Combination of Mps1 inhibition and docetaxel induces earlier spindle multipolarity, cellular pleomorphism and karyotypic copy number variations. **a** Representative images of anaphases from tumour tissue sections immunostained for S10 phosphorylated histone H3, and DNA counterstained with DAPI. Arrowheads highlight the abnormalities scored in each phenotype. **b** Quantification of chromosome missegregations in the tumours after 48 and 72 h of treatment with the indicated drugs. Bars represent means and error bars SD (*n* = 2), statistically different means are indicated with asterisks, *Α* < 0.05 (Dunnett’s multiple comparison test). **c** Quantification of anaphases with multipolar spindles (more than two Υ-tubulin poles) in the tumour slides. Bars represent the mean, error bars represent the 95% C.I. **d** Percentage of nuclear pleomorphism observed in the histopathological analysis of the treated tumours. **e** Quantification of caspase 3-positive cells per field in the different treatment conditions after 24, 48 and 72 h of drug exposure. Bars represent means and error bars standard error of the mean (*n* = 2), asterisk indicates statistically different values relative to the vehicle within each time point (Tukey’s multiple comparisons test). **f** Schematic representation of the effect of exposure to Mps1 inhibitors, taxanes or the combination of both in mitosis progression and cell fate

To better understand the mechanism of action, we repeated the same intervention studies using 10 mg/kg Cpd-5 and 12.5 mg/kg docetaxel, but this time the tumours were harvested during treatment (24, 48, 72 h and 1 week). Chromosome missegregations were observed in approximately one-third of the cell divisions in the vehicle-treated tumour (Fig. 5a, b), confirming previous observations that the BRCA1^−/−^;TP53^−/−^ tumours are CIN.^39^ Exposure to Cpd-5 alone did not have a significant effect on the level of missegregations in these tumours, while treatment with docetaxel led to a minor increase (Fig. 5b). Combination of both drugs gave rise to a major increase in segregation defects without changing the pattern of missegregations (Fig. 5b), but due to a rise in cell divisions with multipolar spindles (Fig. 5b, c). While the vehicle- and Cpd-5-treated samples displayed marginal values of multipolarity (~3%), the tumours treated with docetaxel alone already presented an increase to 20% of multipolar spindles after 72 h of treatment (Fig. 5c). This increase was much more prominent in the tumours treated with the drug combination: 48 h after treatment, we observed 20% of multipolar anaphases, and after 72 h, these levels increased to 52%. Importantly, the multipolarity was invariably accompanied by segregation defects. Histologically, the vehicle- and Cpd-5-treated tumours did not present dramatic changes over the course of treatment (Table 1). In accordance with the increased multipolarity and missegregations, the tumour samples treated with docetaxel alone showed an increase in nuclear pleomorphism. The combinatorial regimen accelerates the onset of nuclear pleomorphism as compared to the single treated samples (Fig. 5d), again indicating that the combination increases cell division errors in the tumours. In addition, the samples treated with the drug combination presented higher levels of apoptosis at 24 and 48 h after treatment, as quantified by the number of caspase 3-positive cells (Fig. 5e).

**Table 1:** Summary of the histopathological findings of the implanted BRCA1^−/−^;TP53^−/−^ tumours collected in different time points after treatment

Treatment	Time point	Histopathology
Vehicle	24 h	Carcinomatous lesions with multiple areas of necrosis and haemorrhages.
	48 h	Carcinomatous lesions.
	72 h	Carcinomatous lesions with large areas of necrosis and haemorrhages.
	1 Week	Carcinomatous lesions with areas of necrosis and areas of squamous cell metaplasia.
10 mg/kg Cpd-5	24 h	Carcinomatous lesions with necrosis and haemorrhages.
	48 h	Carcinomatous lesions.
	72 h	Carcinomatous lesions with necrosis.
	1 Week	Carcinomatous lesions.
12.5 mg/kg docetaxel	24 h	Carcinomatous lesions.
	48 h	Carcinomatous lesions with some necrosis.
	72 h	Carcinomatous lesions.
	1 Week	Carcinomatous lesions with large areas of pleomorphism.
12.5 mg/kg docetaxel+10 mg/kg Cpd-5	24 h	Carcinomatous lesions with necrosis.
	48 h	Carcinomatous lesions with large areas of necrosis.
	72 h	Carcinomatous lesions with pleomorphism.
	1 Week	Carcinomatous lesions with pleomorphism.

Taken together, our results indicate that the treatment with clinically relevant doses of docetaxel and Mps1 inhibitors increases CIN in a murine model of triple-negative breast cancer (TNBC) that results in more and longer cycles of tumour regression that culminate in increased overall survival.

## Discussion

The main limitation for the application of Mps1 inhibitors in vivo has been the toxicity in the gut and bone marrow, which limits the amount of inhibitor that can be used in cancer treatment.^25^ To circumvent excessive toxicity, the combination of Mps1 inhibitors with taxanes has been explored. This strategy was proven to be effective in both tissue culture^14,25,30,33^ and in vivo;^14,25,32^ however, exactly how this regimen induces tumour cell death in vivo has not been explored.

Using a genetically engineered mouse strain that carries the C577Y mutation in the kinase domain of Mps1, we could show that all toxic effects of Cpd-5 depend on inhibition of Mps1. Thus, our uniquely engineered mouse model fully validates target engagement of Cpd-5 in vivo and demonstrates that the specific effects induced by administration of Cpd-5 are solely due to Mps1 kinase inhibition. With this novel mouse model, one can easily ascertain the specificity of Mps1 inhibitors in vivo. This can be of particular interest when considering novel combinatorial therapies, like the recently proposed synergy with immune checkpoint inhibitors.^43^

In an effort to identify a biomarker for the efficacy in combining paclitaxel and Mps1 inhibitors, we screened a panel of breast cancer cell lines and correlated the intrinsic amount of chromosome segregation errors or the expression levels of Mps1 with the synergy scores (Fig. S1). While there was no clear association between these criteria and the response to the drug combination, we could observe that cell lines expressing higher levels of Mps1 were the ones more resistant to paclitaxel (Fig. S1F). This way, we conclude that although the levels of Mps1 kinase predict the sensitivity to taxanes,^41^ they are not a marker for the combinatorial therapy with paclitaxel and Cpd-5.

The ultimate goal of this study was to understand the mechanism underlying the in vivo drug synergy between docetaxel and Cpd-5. Taxanes are standard of care therapy for TNBC, and the treatment with docetaxel is sufficient to drive tumour regression in a TNBC mouse model, but ultimately the tumours relapse and become resistant.^40^ We demonstrate that combining clinically relevant doses of docetaxel with Cpd-5 results in a clear survival benefit, without additional signs of toxicity in the gut and bone marrow. Previous studies, including one from our own lab, have demonstrated that docetaxel and Mps1 inhibitors act synergistic in vivo, and have shown that the treatment combination results in higher CIN.^14,25,32^ Our study provides the first in vivo evidence that the combination of Cpd-5 and docetaxel results in an earlier and more prominent increase in multipolar cell divisions in the tumour, followed by enhanced apoptosis (Fig. 5e). While we cannot ascertain whether tumour cell death occurs through mitotic catastrophe or in the following cell cycle, the different onsets in cellular pleomorphism (Fig. 5d) and cell death (Fig. 5e) after treatment combination suggest that some cells die in mitosis, while the ones that escape a mitotic catastrophe become pleomorphic. This way, we demonstrate that combining a spindle poison with a SAC inhibitor can promote further segregation errors by promoting multipolar cell divisions.

Previous studies have shown that clinically relevant concentrations of paclitaxel induce spindle multipolarity and chromosome missegregations.^44^ Indeed, the serum and intracellular concentrations of docetaxel in the tumours are comparable to the ones in patients (see ref.^44^ and Tables S6 and S7), and we observed an increase in multipolar anaphases after 72 h of exposure to 12.5 mg/kg of docetaxel (Fig. 5c). Notably, the percentage of multipolar cell divisions was greatly increased by the inhibition of Mps1 with Cpd-5. These higher levels of multipolar cell division translated into premature development of nuclear pleomorphism in the mice treated with Cpd-5 and docetaxel and a more persistent induction of tumour cell apoptosis. In addition to the changes in nuclear morphology, the karyotype of the tumours was also altered upon treatment. Copy number analysis of the tumours after prolonged exposure to Cpd-5, docetaxel or both drugs revealed profound changes in CNV in the tumours treated with the double combination, but not in the single treatments. Therefore, we can conclude that the treatment combination induces higher levels of CIN.

Taken together, our results show that the inhibition of Mps1 kinase together with the exposure to clinically relevant doses of taxanes enhances CIN in BRCA1^−/−^;TP53^−/−^ mammary tumours. Taxane treatment induces multipolarity in the mitotic cells, and the presence of an Mps1 inhibitor pushes these cells into anaphase, thus promoting massive chromosome missegregations (Fig. 5f). In some cells, the amount of missegregations will prevent normal mitotic exit, thereby resulting in polyploid cells or mitotic cell death. The remaining population will successfully complete mitosis but give rise to aneuploid progeny. The balance of the chromosome content will dictate whether the resulting daughter cells will survive or die. Of course, we cannot exclude that the combination of Mps1 inhibitors and taxanes can also perturb other pathways, which might enhance tumour cell death. As a disclaimer, we have carefully inspected the tumour morphology after treatment combination and could not identify any consistent alterations apart from the nuclear pleomorphism (Table 1). Therefore, our data are most consistent with a mechanism involving enhanced CIN that causes the combination of Mps1 inhibitors and taxanes to be more effective. This suggests that the extent to which this combination can alter CIN status could be a good predictor of response for this combinatorial therapy that is currently in clinical trials for solid tumours.

## Electronic supplementary material

Supplementary Figure 1

Supplementary Figure 2

Supplementary Figure 3

Supplementary Figure 4

Supplementary Figure 5

Supplementary Figure 6

Supplementary Materials and Methods

Supplementary Tables
