# Supplementary material for: Mps1 inhibitors synergise with low doses of taxanes in promoting tumour cell death by enhancement of errors in cell division
Source: Br J Cancer. 2018 May 8;118(12):1586–95. doi: 10.1038/s41416-018-0081-2 (PMC6008333; doi:10.1038/s41416-018-0081-2)
Supplement: Supplementary file 7 — Supplementary Materials and Methods [file 41416_2018_81_MOESM7_ESM.docx]

**Mps1 inhibitors synergize with low doses of taxanes in promoting tumour cell death by enhancement of errors in cell division**

A.R.R. Maia^1^, S. Linder^1†^, J.Y. Song^2^, C. Vaarting^1^, U. Boon^3^, C.E.J. Pritchard^4^, A. Velds^5^, I.J. Huijbers^4^, O. van Tellingen^6^, J. Jonkers^3^, R.H. Medema^1^*

**Supplementary Materials and Methods**

**Study Design**

The goal of this study was to investigate how the combination of docetaxel and a Mps1 inhibitor (Cpd-5) increases the overall survival in mice with triple-negative breast cancer. Our current and previous results have shown that the combination of Mps1 inhibitors with docetaxel enhances the cytotoxic activity of the taxanes, but the mechanism behind this drug synergy is unknown. We reasoned that the genomic instability present in the *K14cre;Brca1^f/f^;tp53^f/f^* tumour model^1^ renders an increased sensitivity to agents that severely alter the chromosome content of these tumours. For this purpose, eight-week-old FVB wild-type female mice were defined as the experimental unit for the *in vivo* experiments. For the MTD studies, animals were allocated in cages according to treatment group: vehicle, 5, 10, 15 and 25 mg/kg Cpd-5, 25 mg/kg docetaxel, 5 mg/kg Cpd-5 with 25 mg/kg docetaxel, 10 mg/kg Cpd-5 with 25 mg/kg docetaxel, 10 mg/kg Cpd-5 with 12.5 mg/kg docetaxel. For the long-term intervention studies, we followed a randomized experimental design: before transplantation, mice were allocated according to internal identification numbers. Treatments were initiated once the tumour reached the volume of 200 mm^3^ and mice were randomly assigned to the different tumour groups. This way, the same cage contained animals from different treatment groups. Mice were divided in five single treatment groups: vehicle, 5 and 10 mg/kg Cpd-5, 25 and 12,5 mg/kg docetaxel; and two combination groups: 25mg/kg docetaxel with 5 mg/kg Cpd-5 and 12.5 mg/kg docetaxel with 10 mg/kg Cpd-5. The treatments and handling were preferentially performed in the morning. Due to the specific type of treatments, the researchers were aware of which treatment group the mouse belonged to. For the time point intervention studies, we followed a similar protocol. But instead of evaluating the tumour response to treatment, the tumours were collected in specific time points: 24, 48, 72 hours and one week after treatment. For these studies, mice were treated with vehicle, 10 mg/kg Cpd-5, 12.5 mg/kg docetaxel and 12,5 mg/kg docetaxel with 10 mg/kg Cpd-5.

For the animal studies, data collection was interrupted according to the humane endpoints previously established with the Animal Ethics Committee. The primary endpoint for the MTD studies was defined at a weight loss of 20% or more (measured weight at first treatment). For the intervention studies, the primary endpoint was set once the tumours reached a volume bigger than 1500 mm^3^ or 20% or more of weight loss. For the long-term intervention studies, four experiments were performed with different tumour donors (MDR proficient and deficient). For the time point intervention studies, one experiment was done with a MDR proficient tumour donor. The number of animals and group treatments was equally defined for each tumour donor.

The animals in which tumours failed to grow after the transplantation protocol were not reported in the manuscript since they were not included in treatment or data analysis. In the intervention studies, all mice sacrificed due to drug toxicity were considered outliers and excluded from analysis (related to Fig. 3). This decision was made posteriorly to data acquisition since these animals were all included in treatment.

After conclusion of the experiments, data was divided in the different treatment groups and analysed according to those. Histo-pathological analyses of the MTD and intervention studies were performed blindly after grouping the slides according to treatment.

**Cell proliferation assays**

Cells were plated on 96-well plates (BD Biosciences) and drugs were added on day 1 using a Digital Dispenser (Tecan Männedorf). On day 8, cells were fixed with 96% methanol and stained with 0,1% crystal violet. Plates were scanned and analysed with ImageJ software (NIH) and relative cell survival plots were generated with Prism 7 (GraphPad). Drug synergy was calculated with MacSynergy™ II software ^2^.

**Flow cytometric cell cycle analysis**

Cells were harvested and fixed in 70% ethanol after 24 h, 48 h or 72 h of drug treatment. Samples were stained with 1 μg/mL DAPI and treated with 0.25 mg/mL RNase A (Sigma), directly before the flow cytometric analysis. For cell cycle analysis, 30,000 events per samples were analysed in a BD LSRFortessa analyser (BD Biosciences) using the 405 and 488 nm lasers. Data was analysed with FlowJo software.

**Live cell imaging**

KB1P-B11 cells were platted on LabTek dishes and two hours before imaging, the media was changed to Leibovitz L15 CO_2_‐independent cell culture medium (Gibco). Mitotic progression was followed by adding SiR-DNA (Spirochrome) and drugs to the cells, that were imaged as previously described ^3^. Cell death upon drug treatment was scored live with the probe TO-PRO-3 Iodide (Life Technologies). For this purpose, cells were imaged every 15 minutes in a heated chamber of 37 °C using a 20x NA 0.95 air objective on an IX71 microscope (Olympus), controlled by SoftWoRx 6.0 software (Applied Precision).

**Chromosome spreads**

Cells were treated with the indicated drugs for 24 h, 48 h or 72 h, followed by washout of the compounds and incubation with 250 ng/μL nocodazole (Sigma) for 2 h at 37°C. Cells were harvested by mitotic shake off and incubated at 37°C for 10 min in 75 mM KCl solution, and fixed in methanol and acetic acid (3:1) and stained with 1 μg/mL DAPI. Cells were added dropwise on microscope slides and mounted with Vectashield (Vector Laboratories). For analysis, chromosome spreads were imaged using a 100x NA 1.4 oil objective (Olympus) on an IX71 microscope. Images were acquired using a DeltaVision Elite system equipped with a sCMOS camera and controlled by SoftWoRx software. Image analysis was carried out using ImageJ and plots were performed with Prism 7 (GraphPad).

**CRISPR/Cas-9-mediated genome editing**

The guide RNA (5’- GAAACATTGACCTAAATAGT -3’) was designed according to the sequence downstream of MPS1-C577Y and cloned into a pX330 vector (Addgene, ^4^). KB1P-B11 cells were co-transfected with the pX330/gRNA and an oligonucleotide including the C577Y mutation (5’-GTCTGTTTATCTTTAATTGCAGTGAAATCACCGAGCAGTACATCTACATGGTAATGGAATATGGAAACATTGACCTAAATTCCTGGCTTAAAAAGAAAAAATCCATCAATCCATGGGAACGCAAGAGCTACTGGAAAAACATG-3’, Integrated DNA Technologies) using FuGENE HD (Promega). Two days post-transfection, cells were selected with 100 nM Cpd-5 for 4 weeks.

For the generation of the resistant mouse strains, zygotes of FVB/NrJ mice were injected with *in vitro* transcribed Cas9 mRNA, the guide RNA for Mps1 kinase, and the oligo to introduce the C577Y mutation and three silent mutations^5^. To control for the process of gene editing, a control strain containing only the silent mutations was generated. After two generations of backcrossing, the pups were screened by PCR and Sanger sequencing.

**Genotyping**

Control and resistant strains were genotyped by PCR with the primer sets: Forward - 5’ GCAACACAGTGATAAGATCATCC 3’; Reverse - 5’ GATTTTTTCTTTTTAAGCCAGGA 3’. For Sanger sequencing, the following primer sets were used: Forward - 5’ GTGGGCATTTATTGTTTTGTTGTG 3’; Reverse - 5’ CTGTGCTTGTTTGTGTTCTGTAGC 3’.

**Histopathology**

Formalin-fixed paraffin-embedded (FFPE) tumours were sectioned at 4 μm and stained with hematoxilin and eosin for histological evaluation according to standard procedures. Active Caspase 3 (Cell Signaling; 9661L) was detected by immunohistochemistry and quantified as the number of labelled cells per field of view.

For the scoring of missegregations, sections were stained using citrate antigen retrieval protocol with phospho-Histone H3 (Ser10) (06-570, Millipore), and Alexa 568 anti-rabbit (A-11011**,** Life Technologies). Multipolar anaphases were scored by staining the slides with gamma-tubulin antibody (clone GTU-88, Sigma) and Alexa 568 anti-mouse (A-11004**,** Life Technologies). DNA was counterstained with DAPI.

**Low coverage copy number sequencing and data processing**

Samples for copy number variation sequencing were processed and analysed as described in Schouten *et al*^6^.

**Pharmacokinetic Studies**

Compound 5 and docetaxel were measured by LC-MS/MS. The system comprised an UltiMate 3000 LC System (Dionex, Sunnyvale, CA) and an API4000 mass spectrometer (Sciex, Framingham, MA). NMS-P715 and docetaxel-d9 were used as internal standards. Tumours were homogenized using a FastPrep®-24 (MP-Biomedicals, NY) in 1% (w/v) bovine serum albumin in water and diluted 10-fold in human plasma. Plasma samples and tumour homogenates (100 ul) and internal standard (50 ul) were mixed with 1 ml diethyl ether. The organic layer was separated, dried and reconstituted in 100 ul of methanol:water (20;80; v/v). Samples (50 ul) were subjected to HPLC using a Securityguard C18 pre-column (Phenomenex, Utrecht, The Netherlands) coupled to a 100 x 2 mm (ID) ZORBAX Extend-C18 column (Agilent, Santa Clara, CA). Chromatographic separation involved a 5-minute gradient from 20% to 95% B (mobile phase A: 0.1% formic acid in water (v/v) and mobile phase B: methanol). 95% B was maintained for 3 min followed by re-equilibration at 20% B. Multiple reaction monitoring was performed at 581.1/404.3 (Compound 5), 677.3/563.2 (NMS-P715), 808.5/527.3 (docetaxel) and 817.5/527.2 (docetaxel-d9). Analyst^®^ 1.6.2 software was used for system control and data analysis.

**Statistical analysis**

Relative cell survival plots were generated with Prism 7 software (GraphPad). Kaplan-Meier survival curves were analysed by log-rank test, with a level of significance of p<0.05. Overall survival was calculated as time from first treatment to death from any cause. Statistical analysis of chromosome segregation and cell death in cell lines was performed with GraphPad Prism v7.0b as indicated in the figures.

**Supplementary references**

1. Liu X, Holstege H, van der Gulden H, Treur-Mulder M, Zevenhoven J, Velds A, *et al.* Somatic loss of BRCA1 and p53 in mice induces mammary tumors with features of human BRCA1-mutated basal-like breast cancer. *Proc Natl Acad Sci U S A* 2007; **104**: 12111-12116.

2. Prichard MN, Shipman C, Jr. A three-dimensional model to analyze drug-drug interactions. *Antiviral Res* 1990; **14**: 181-205.

3. Maia AR, de Man J, Boon U, Janssen A, Song JY, Omerzu M, *et al.* Inhibition of the spindle assembly checkpoint kinase TTK enhances the efficacy of docetaxel in a triple-negative breast cancer model. *Ann Oncol* 2015; **26**: 2180-2192.

4. Cong L, Ran FA, Cox D, Lin S, Barretto R, Habib N, *et al.* Multiplex genome engineering using CRISPR/Cas systems. *Science* 2013; **339**: 819-823.

5. Huijbers IJ, Del Bravo J, Bin Ali R, Pritchard C, Braumuller TM, van Miltenburg MH, *et al.* Using the GEMM-ESC strategy to study gene function in mouse models. *Nat Protoc* 2015; **10**: 1755-1785.

6. Schouten PC, Grigoriadis A, Kuilman T, Mirza H, Watkins JA, Cooke SA, *et al.* Robust BRCA1-like classification of copy number profiles of samples repeated across different datasets and platforms. *Mol Oncol* 2015; **9**: 1274-1286.

**Supplementary figure legends**

**Figure S1 –** Synergistic activity between paclitaxel and Mps1 inhibitors in mammary cell lines. **A)** Relative survival plots of the KB1P-B11 cell line treated with paclitaxel and the Mps1 inhibitor BAY 1217389. **B)** Relative survival plots of a panel of breast cancer cell lines treated with paclitaxel with and without additional Cpd-5 treatment. Shown is the average of three independent experiments, with the respective standard deviations. **C)** Quantification of total chromosome missegregations in the breast cancer cell lines. **D)** Western blot analysis of Mps1 and Cyclin B1 levels in the breast cancer panel. **E)** Correlation plots between the synergy scores and the percentage of missegregations (left), Mps1 levels (right). The values indicate the Pearson correlation values. **F)** Correlation plots between the Mps1 levels and paclitaxel IC_50_s. The values indicate the Pearson correlation values.

**Figure S2** – Combination of paclitaxel and Mps1 inhibition leads to chromosomal instability in the KB1P-B11 cell line. **A)** Representative examples of time-lapse imaging (phase contrast) of a dividing KB1P-B11 cell undergoing cytokinesis failure. **B)** Representative examples of time-lapse imaging of a KB1P-B11 cell labelled with SiR-DNA slipping out mitosis. Arrowheads indicate the abnormalities present in each class and the number show the time in minutes from nuclear envelope breakdown (time 0’). Images are related to the scored polyploid phenotypes shown in Fig. 1D. **D)** Histograms representing the quantifications of mitotic chromosome spreads of KB1P-B11 cells untreated or treated with 30 nM Cpd-5, 3 nM paclitaxel, or a combination of both for 24 h, 48 h or 72 h (20 cells per condition).

**Figure S3** – Synergy between paclitaxel and Cpd-5 is Mps1 kinase dependent. A) Characterization of the KB1P-B11-C577Y cell line regarding its sensitivity to Cpd-5 in comparison to the parental KB1P-B11 cell line. **B)** Representative colony formation assay of KB1P-B11-C577Y cells treated for 7 days with increasing concentrations of paclitaxel and/or Cpd-5. **C)** Relative survival plots of paclitaxel treated cells with and without additional Cpd-5 treatment. Shown is the average of three independent experiments, with the respective standard deviations. **D)** 3D synergy plots of the drug combination between Cpd-5 and paclitaxel in KB1P-B11-C577Y cell line.

**Figure S4** – Compound 5 toxicity in the bone marrow is absent in the resistant mice. **A, B)** Representative images of H&E stainings of the bone marrow of control mice, TTK_C577Y (3) **(A)** and resistant mice, TTK_C577Y (1) **(B)** treated with vehicle (left panels) or 25 mg/kg Cpd-5 (right panels).

**Figure S5** – Growth curves of the tumours in treatment. **A)** Individual tumour growth curves of mice treated with vehicle, 5 or 10 mg/kg Cpd-5 (n = 10, 11 and 11, respectively). **B)** Individual tumour growth curves of mice treated with 25 mg/kg docetaxel alone, or in combination with 5 mg/kg Cpd-5 (n = 11 and 14, respectively). **C)** Individual tumour growth curves of mice treated with 12.5 mg/kg docetaxel alone, or in combination with 10 mg/kg Cpd-5 (n = 10 and 17, respectively). **D)** H&E staining of the mammary fat pad of the mouse in remission. Right figure shows a higher magnification of the squared area in the left panel to highlight the quiescent tumour cells that remained in the mammary fat pad.

**Figure S6** – Copy number variation of the tumour samples after treatment. Genome wide karyotype quantification by CNVseq of the vehicle treated tumour, and the tumours treated with 10 mg/kg Cpd-5, 12,5 mg/kg docetaxel and with the combination of both drugs.
