# Supplementary material for: Mps1 inhibitors synergise with low doses of taxanes in promoting tumour cell death by enhancement of errors in cell division
Source: Br J Cancer. 2018 May 8;118(12):1586–95. doi: 10.1038/s41416-018-0081-2 (PMC6008333; doi:10.1038/s41416-018-0081-2)
Supplement: Supplementary file 8 — Supplementary Tables [file 41416_2018_81_MOESM8_ESM.docx]

| **Table S1 –** IC_50_ of Paclitaxel in the panel of mammary cell lines. Values represent the average IC_50_ and respective standard error of three independent experiments. | | | | | | |
| --- | --- | --- | --- | --- | --- | --- |
| **Cpd-5 (nM)** | 0 | 10 | 20 | 30 | 40 | 50 |
| **Paclitaxel IC_50_ (nM)**  **KB1P-B11** | 6.10  ± 0.13 | 5.91  ± 0.17 | 3.83  ± 0.25 | 2.01  ± 0.19 | 0.686  ± 0.729 | 3.16  ± 0.73 |
| **Paclitaxel IC_50_ (nM) KP3** | 19.86  ± 0.25 | 9.89  ± 0.26 | 5.78  ± 0.42 | 2.39  ± 0.27 | 1.72  ± 0.14 | 0.40  ± 0.47 |
| **Paclitaxel IC_50_ (nM) MCF 10A** | 2.10  ± 0.023 | 2.01  ± 0.054 | 1.76  ± 0.097 | 1.20  ± 0.12 | 0.63  ± 0.29 | -1.22  ± 1.97 |
| **Paclitaxel IC_50_ (nM) MCF7** | 0.79  ± 0.14 | 0.52  ± 0.16 | -0.32  ± 0.38 | -0.21  ± 0.68 | -0.038  ± 2.06 | 2.87  ± 9.14 |
| **Paclitaxel IC_50_ (nM) MDA-MB-361** | 5.70  ± 0.52 | 6.04  ± 0.54 | 5.95  ± 0.49 | 5.80  ± 0.46 | 5.91  ± 0.46 | 5.75  ± 0.45 |
| **Paclitaxel IC_50_ (nM) MDA-MB-468** | 1.43  ± 0.13 | 1.36  ± 0.13 | 0.95  ± 0.10 | 0.57  ± 0.067 | 0.21  ± 0.26 | -0.79  ± 1.69 |
| **Paclitaxel IC_50_ (nM) MDA-MB-231** | 0.61  ± 0.19 | 0.25  ± 0.28 | -0.22  ± 0.56 | ND | ND | 1.69  ± 0.30 |
| **Paclitaxel IC_50_ (nM) SK-BR-3** | 2.33  ± 0.064 | 2.35  ± 0.11 | 2.20  ± 0.084 | 2.05  ± 0.092 | 1.64  ± 0.092 | 1.31  ± 0.11 |

| **Table S2 –** Genotypes of the breast cancer cell lines ({Smith, 2017 #60}) | | | | | | | | |
| --- | --- | --- | --- | --- | --- | --- | --- | --- |
| **Cell Line** | MCF 10A | MCF7 | MDA-MB-361 | MDA-MB-468 | MDA-MB-231 | SK-BR-3 | KP3 | KB1P-B11 |
| **Subtype** | Basal B | Luminal A | Luminal B | Basal A | Basal B | HER2 |  | Basal |
| **ER** | - | + | + | - | - | - | - | - |
| **PR** | - | + | + | - | - | - |  | - |
| **HER2** | - | - | + | - | - | + |  | - |
| **BRCA1 status** | Wild type | Wild type | Wild type | Wild type | Wild type | Wild type | Knock out | Knock out |
| **TP53 status** | Wild type | Wild type | Mutant | Mutant | Mutant | Mutant | Knock out | Knock out |

| **Table S3 –** Synergy scores of the drug combination of Cpd-5 and Paclitaxel in a panel of breast cell lines. | | | | | | | | |
| --- | --- | --- | --- | --- | --- | --- | --- | --- |
| **Cell Line** | MCF 10A | MCF7 | MDA-MB-361 | MDA-MB-468 | MDA-MB-231 | SK-BR-3 | KP3 | KB1P-B11 |
| **Synergy Score** | 6.75 | 0 | 2.14 | 3.48 | 70.75 | 0 | 18.32 | 74.56 |

| **Table S4** – Summary of the histo-pathological findings of the tissues from the MTD study in the control mice. | | | | |
| --- | --- | --- | --- | --- |
| **Treatment** | **Jejunum/Ileum** | **Colon** | **Spleen** | **Bone marrow** |
| Vehicle | No alterations | No alterations | No alterations | No alterations |
| 25 mg/kg Cpd-5 | Increased apoptotic and mitotic cells in the crypts of jejunum. The lesions are severer in ileum showing reduction and depletion of the crypts, degeneration of the covering epithelia and  lymphocytic infiltrations. | Lesions in cecum and proximal colon similar to the ones described in ileum. | Low hematopoiesis. | Marked reduction of the cellularity of all cell lineages in the bone marrow of sternum. |

| **Table S5 –** IC_50_ of Paclitaxel in KB1P-B11 cell line with the C577Y mutation. Values represent the average IC_50_ and respective standard error of three independent experiments. | | | | | | |
| --- | --- | --- | --- | --- | --- | --- |
| **Cpd-5 (nM)** | 0 | 10 | 20 | 30 | 40 | 50 |
| **Paclitaxel IC_50_ (nM)** | 7.79  ± 0.16 | 7.80  ± 0.13 | 7.89  ± 0. 099 | 6.32  ± 0.14 | 6.24 ±  0.11 | 6.37  ± 0.13 |

| **Table S6 –** Cpd-5 measurements in serum and by tumour weight. Values represent the mean concentration and respective standard deviation from more than two samples (when available). | | | |
| --- | --- | --- | --- |
| **Sample** | **Serum (nM)** | **Tumour (nM)** |  |
| 10 mg/kg Cpd-5 (24h) | 7,3 | 440 |  |
| 10 mg/kg Cpd-5 (24h) | 3,4 | 423 |  |
| 10 mg/kg Cpd-5 (48h) | <1 | ND |  |
| 10 mg/kg Cpd-5 (48h) | < 1 | ND |  |
| 10 mg/kg Cpd-5 (72h) | ND | 440 |  |
| 10 mg/kg Cpd-5 (72h) | 1,4 | 284 |  |
| 10 mg/kg Cpd-5 (72h) | < 1 | ND |  |
| 10 mg/kg Cpd-5 (1 week) | 21,6 | ND |  |
| 12,5 mg/kg docetaxel + 10 mg/kg Cpd-5 (24h) | 2,7 | 234 |  |
| 12,5 mg/kg docetaxel + 10 mg/kg Cpd-5 (24h) | 1,2 | 515 |  |
| 12,5 mg/kg docetaxel + 10 mg/kg Cpd-5 (24h) | < 1 | ND |  |
| 12,5 mg/kg docetaxel + 10 mg/kg Cpd-5 (48h) | < 1 | 291 |  |
| 12,5 mg/kg docetaxel + 10 mg/kg Cpd-5 (48h) | < 1 | ND |  |
| 12,5 mg/kg docetaxel + 10 mg/kg Cpd-5 (72h) | 1,6 | 223 |  |
| 12,5 mg/kg docetaxel + 10 mg/kg Cpd-5 (72h) | < 1 | ND |  |
| 12,5 mg/kg docetaxel + 10 mg/kg Cpd-5 (1 week) | < 1 | ND |  |
| 12,5 mg/kg docetaxel + 10 mg/kg Cpd-5 (1 week) | < 1 | ND |  |

| **Table S7 –** Paclitaxel measurements in serum and by tumour weight. Values represent the mean concentration and respective standard deviation from more than two samples (when available). | | |
| --- | --- | --- |
| **Treatment** | Serum (nM) | Tumour (nM) |
| 12,5 mg/kg docetaxel (24h) | 13,7 | 1171 |
| 12,5 mg/kg docetaxel (24h) | 8,8 | 3886 |
| 12,5 mg/kg docetaxel (24h) | 16,4 | 3564 |
| 12,5 mg/kg docetaxel (48h) | 8,5 | 1648 |
| 12,5 mg/kg docetaxel (48h) | 3,7 | 282 |
| 12,5 mg/kg docetaxel (72h) | 1,7 | 604 |
| 12,5 mg/kg docetaxel (72h) | 1 | 760 |
| 12,5 mg/kg docetaxel (72h) | 1,8 | ND |
| 12,5 mg/kg docetaxel (1 week) | < 1 | ND |
| 12,5 mg/kg docetaxel (1 week) | < 1 | 3052 |
| 12,5 mg/kg docetaxel (1 week) | < 1 | ND |
| 12,5 mg/kg docetaxel + 10 mg/kg Cpd-5 (24h) | < 1 | ND |
| 12,5 mg/kg docetaxel + 10 mg/kg Cpd-5 (24h) | 5 | 4424 |
| 12,5 mg/kg docetaxel + 10 mg/kg Cpd-5 (24h) | 18,6 | 1811 |
| 12,5 mg/kg docetaxel + 10 mg/kg Cpd-5 (48h) | 5,2 | 1795 |
| 12,5 mg/kg docetaxel + 10 mg/kg Cpd-5 (48h) | 5 | 1557 |
| 12,5 mg/kg docetaxel + 10 mg/kg Cpd-5 (72h) | ND | 1176 |
| 12,5 mg/kg docetaxel + 10 mg/kg Cpd-5 (72h) | 1,6 | 2855 |
| 12,5 mg/kg docetaxel + 10 mg/kg Cpd-5 (1 week) | < 1 | 881 |
| 12,5 mg/kg docetaxel + 10 mg/kg Cpd-5 (1 week) | < 1 | ND |
